# Physiological and Aberrant γ-Globin Transcription During Development

**DOI:** 10.3389/fcell.2021.640060

**Published:** 2021-04-01

**Authors:** Gloria Barbarani, Agata Labedz, Sarah Stucchi, Alessia Abbiati, Antonella E. Ronchi

**Affiliations:** Dipartimento di Biotecnologie e Bioscienze, Università di Milano-Bicocca, Milano, Italy

**Keywords:** globin genes, transcription factors, hereditary persistence of fetal hemoglobin, juvenile myelomonocytic leukemia, erythropoiesis

## Abstract

The expression of the fetal Gγ- and Aγ-globin genes in normal development is confined to the fetal period, where two γ-globin chains assemble with two α-globin chains to form α_2_γ_2_ tetramers (HbF). HbF sustains oxygen delivery to tissues until birth, when β-globin replaces γ-globin, leading to the formation of α_2_β_2_ tetramers (HbA). However, in different benign and pathological conditions, HbF is expressed in adult cells, as it happens in the hereditary persistence of fetal hemoglobin, in anemias and in some leukemias. The molecular basis of γ-globin differential expression in the fetus and of its inappropriate activation in adult cells is largely unknown, although in recent years, a few transcription factors involved in this process have been identified. The recent discovery that fetal cells can persist to adulthood and contribute to disease raises the possibility that postnatal γ-globin expression could, in some cases, represent the signature of the fetal cellular origin.

## Erythropoiesis During Development

During mammalian development, hematopoiesis is regulated both spatially and temporally: it begins in the yolk sac, it goes through a transitory phase in the fetal liver and then is definitively established in the thymus and bone marrow (Dzierzak and Bigas, 2018). The first erythroid precursors emerge from the yolk sac as soon as the embryo grows too big to be supplied with oxygen by diffusion and give rise to primitive erythroid cells (EryPs). These cells are released in the bloodstream when they are still nucleated and are characterized by the expression of embryonic globins. A second wave of cells migrating into the fetal liver from the yolk sac support fetal hematopoiesis until birth, in the interval between primitive and definitive hematopoietic stem cell (HSC)-dependent hematopoiesis. Of interest, recent studies suggest that in mouse, this second fetal transient hematopoietic wave of yolk sac-derived erythro-myeloid progenitors (EMPs) may persist postnatally (Epelman et al., 2014; Gomez Perdiguero et al., 2015). HSCs arise from the hemogenic endothelium of the embryonic aorta-gonad-mesonephros (AGM), the vitelline and umbilical arteries, and from the placenta (Dzierzak and Philipsen, 2013; Lacaud and Kouskoff, 2017; Dzierzak and Bigas, 2018). These cells migrate first into the FL and then to the bone marrow (BM)—their long-term adult resident location—where they will last throughout life and will generate all types of blood cells, including erythrocytes (Orkin and Zon, 2008; Palis, 2014; Dzierzak and Bigas, 2018).

The different types of erythroid cells produced at the different hematopoietic stages have many common characteristics, including the main steps of progressive differentiation and maturation from early progenitors to erythroblasts and finally to red blood cells (RBCs) (Kina et al., 2000; Aisen, 2004; Chen et al., 2009; Baron, 2013; Palis, 2014). However, importantly, they can be in part distinguished by differences in cell morphology and in the expression of embryo/fetal vs. adult globins.

## Globin Genes

**FIGURE 1:**
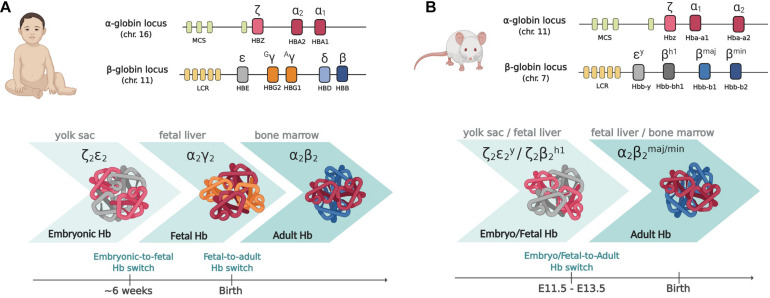
The globin gene loci in humans **(A)** and mice **(B)**. Upper panels: the globin gene loci. MCS (multispecies conserved sequences) and LCR (locus control region) are the distal enhancers coordinating the developmental expression of the genes within the cluster. Lower panels: the different types of globin chains expressed at the different developmental stages and the corresponding sites of hematopoiesis are shown. Please note that whereas in humans two subsequent switching events take place, in mouse, as the majority of mammals, there is only one switch from embryo/fetal to adult genes, occurring early in development. Created with BioRender.com.

In humans, the α-globin cluster contains three functional genes: the embryonic, HBZ (ζ-globin) and the two fetal/adult HBA2 and HBA1 duplicated genes (α2- and α1-globin) (Stamatoyannopoulos, 2005). The β-globin cluster contains five active genes: the embryonic HBE (ε-globin) gene, the two highly homologous fetal HBG2 and HBG1 genes (Gγ- and Aγ-globin, respectively) and the two adult HBD and HBB genes (δ- and β-globin, the latter accounting for about 98% of adult β-like globin) (Figure 1). Each locus is under the control of a set of distal enhancers (Grosveld et al., 1987; Higgs et al., 1990). The genes contained in the α-globin and β-globin loci are sequentially expressed in a stage-specific manner that maintains the 1:1 ratio between the α-like and β-like globin chains, in a process known as “hemoglobin switching” (Forget, 1990; Stamatoyannopoulos, 2005; Sankaran et al., 2010a).

Interestingly, the presence of fetal-specific (γ) genes and thus of a fetal (γ) to adult (β) globin switch is unique to humans and old-world monkeys: most species, including mice, have only one switch, from embryo/fetal to definitive globin genes expression, occurring early in development (Stamatoyannopoulos, 1991; Sankaran et al., 2010a; Philipsen and Hardison, 2018; Figure 1). In mice transgenic for the human β-globin locus, the switching of human globin genes parallels the switching of mouse genes, with γ genes being switched off between E11.5 and E13.5, together with εγ and βh1 mouse embryo/fetal genes (Strouboulis et al., 1992; Peterson et al., 1995). Each developmental switch is accompanied by a profound chromatin remodeling within the loci: the interaction (“loop”) between the promoter of the gene active at a given time with the common distal enhancers [locus control region (LCR)] is progressively favored, with inactive globin genes being looped out (Tolhuis et al., 2002; Palstra et al., 2003). Whereas only EryPs of yolk sac origin expresses embryonic ζ-, human ε- and mouse εy-globin genes, the other globin genes are more promiscuously expressed by cells of different origin. McGrath et al. (2011) showed that the first cells expressing adult globins, prior to the generation of HSC-derived erythroblasts, are indeed the transient population of EMP-derived erythroid cells.

## Physiological and non Physiological γ-Globin Expression

The switching from γ- to β-globin expression is the most intensively studied because the persistence of γ-globin expression in adult stages is a hallmark of a very heterogeneous spectrum of conditions. These can be benign, as in the case of the few F cells (cells expressing HbF) found in normal adults (Boyer et al., 1975) and in HPFH (hereditary persistence of fetal hemoglobin) (Forget, 1998), or associated with disease, such as in response to transient or chronic anemias (Weatherall, 2001) or leukemias (Weatherall et al., 1968; Sheridan et al., 1976). As an additional reason of interest, the ability of reactivating γ-globin in the adult is considered as a possible strategy to cure β-hemoglobinopathies (Wienert et al., 2018). The cause of γ-globin expression in adult cells remains largely unknown and is thought to rely on different mechanisms, both maturational and/or directly related to defects intrinsic to the HBB locus (Zago et al., 1979; Stamatoyannopoulos, 2005). Although these aspects are strictly intertwined, and it is almost impossible to sharply separate them, this review will focus on the latter, in particular on the major transcription factors (TFs) that, by directly binding to the HBB locus, act as selective on/off switches of γ-globin expression in normal and aberrant conditions.

## The Transcriptional Switches of γ-Globin Expression

**FIGURE 2:**
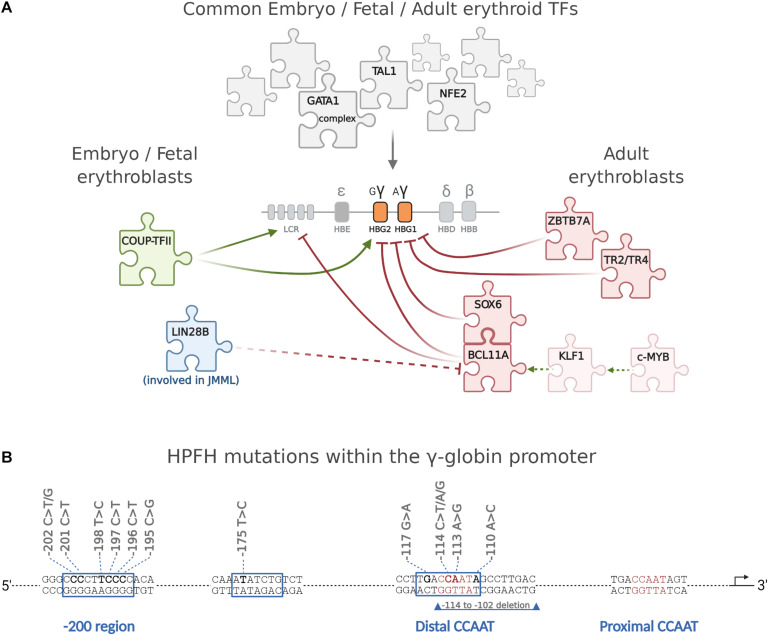
The major transcription factors directly regulating the differential expression of γ-globin during development. **(A)** The expression of γ-globin in embryonic, fetal, and adult cells is regulated by a large common set of ubiquitous (such as, for example, NF-Y) and erythroid-specific transcription factors, the most important of which are GATA1 and its complexes (Love et al., 2014), NFE2, and TAL1. A very small number of TFs, discussed in the text, are instead directly involved in the time-specific expression of γ-globin and in its deregulation when it persists in the adult. c-MYB activates KLF1 that in turn activates BCL11A, which cooperates with SOX6 in repressing γ-globin. ZBTB7A and TR2/TR4 repress γ-globin independently from BCL11A. Of interest, whereas different γ-globin-specific repressors have been identified so far, little is known about early specific activation of embryo/fetal globin genes, and COUP-TFII is the only γ-globin-specific activator identified so far. The oncofetal LIN28B protein, expressed at high levels in JMML cells concomitantly expressing high γ-globin, blocks BCL11A translation. **(B)** Schematic representation of the γ-promoter showing the position of HPFH mutations. Created with BioRender.com.

The observation that the main differentiation and maturation steps leading to RBC formation are common to YS-, EMP-, and HSC-derived erythroblasts (Kina et al., 2000; Aisen, 2004; Chen et al., 2009; Palis, 2014) suggests that these cells may also rely on a common set of transcription factors directing erythroid differentiation and globin gene regulation. Indeed, embryonic/fetal and adult cells share a common set of ubiquitous (such as NF-Y, that binds all globin promoters, although with different affinity (Liberati et al., 1998; Zhu et al., 2012; Martyn et al., 2017)) and erythroid-specific activators/coactivators [first of all GATA1 (Ferreira et al., 2005; Love et al., 2014; Katsumura et al., 2017; Barbarani et al., 2019), NFE2(Gasiorek and Blank, 2015; Kim et al., 2016), KLF1(Perkins et al., 2016), and TAL1 (Kang et al., 2015)]. Moreover, the expression of γ-globin genes in adult cells, as in HPFH, suggests that adult cells represent an environment permissive for the expression of both embryo/fetal and adult globin genes. It is thus likely that the specific timing of γ-globin expression might require specific activators/repressors acting at different times. The number of transcription factors directly involved in the activation/repression of γ-globin transcription is surprisingly small, and their main characteristics are briefly reviewed here below (Figure 2).

## Transcription Factors Affecting the Embryo/Fetal to Adult Switching

### γ-Globin Repressors

BCL11A, also known as CTIP1 (Coup-TFII interacting protein), is a C2H2-type zinc-finger protein (Avram et al., 2000). Alternative splicing generates four major protein isoforms, sharing a common N-terminus: eXtra-Long (XL), Long (L), Short (S), and eXtra-Short (XS) (Liu et al., 2006). BCL11A plays important roles in non-erythroid hematopoietic cells, including B cells (Liu et al., 2003), dendritic cells (Ippolito et al., 2014), and hematopoietic stem cells (HSCs) (Tsang et al., 2015; Luc et al., 2016). Outside hematopoiesis, BCL11A is essential for central nervous system development (Basak et al., 2015; Funnell et al., 2015; Greig et al., 2016) and possibly for the differentiation of other lineages, such as breast (Khaled et al., 2015) and pancreas (Peiris et al., 2018) cells. Its specific role in γ-globin silencing was identified by genome-wide association studies (GWAS) aiming to identify eQTLs (expression quantitative trait loci) associated with high levels of postnatal HbF (Menzel et al., 2007; Lettre et al., 2008; Uda et al., 2008). Its conditional knockout within the erythroid compartment [obtained by disrupting the erythroid-specific BCL11A enhancer (Bauer et al., 2013; Canver et al., 2015)] impairs HbF silencing in adult erythroid cells, without altering erythropoiesis in the mouse (Sankaran et al., 2008, 2010b; Xu et al., 2013). This latter observation made BCL11A-targeted inactivation in erythroid cells a promising approach to reactivate γ-globin in β-hemoglobinopathies (Sankaran et al., 2010a; Wienert et al., 2018; Zeng et al., 2020). Interestingly, in human cells, S and XS isoforms are specific of YS primitive and FL erythropoiesis, whereas XL and L are specific of BM definitive erythropoiesis (Sankaran et al., 2009). Notably, the mouse *Bcl11A* pattern of expression is different, with the XL isoform being already present in FL definitive erythroid cells. This delay in BCL11A-XL in humans could explain the different timing of γ-globin switching in human vs. mouse (Sankaran et al., 2009). The specific γ-globin repression in adult cells is mediated by its binding to the consensus sequence GGTCA, present in several discrete sites within the β-locus (Liu et al., 2018). Among them, the site in the distal CCAAT box region of the γ-promoter, which contains the −117 residue, whose G > A mutation causes HPFH, indeed abolishes BCL11A-XL binding (Martyn et al., 2018).

BCL11A is activated by *KLF1/EKLF* (Borg et al., 2010; Zhou et al., 2010) (Kruppel-Like Factor-1), an erythroid-specific zinc finger TF originally identified because of its ability to bind to CACCC motifs (Miller and Bieker, 1993) and now recognized as a critical regulator of many aspects of erythropoiesis (Perkins et al., 2016). The KLF1 gene knockout in the mouse results in embryonic lethality at around stages E14–E15 due to lethal anemia because of the inability to activate β-globin (Nuez et al., 1995; Perkins et al., 1995) and mutations in the CACCC box of the β-globin promoter that abolish its binding causing thalassemia (Feng et al., 1994). Thus, KLF1 promotes the switching from γ- to β-globin gene expression both directly, by activating the β-globin promoter and indirectly, by activating BCL11A. Finally (Basak et al., 2020), recently demonstrated that the oncofetal protein LIN28B (Piskounova et al., 2011; Shyh-Chang et al., 2013), already known to increase γ-globin expression (Lee et al., 2013, 2015; de Vasconcellos et al., 2014), blocks BCL11A translation. The failure of the above regulatory circuits converging on BCL11A results in elevated HbF.

SOX6 is a HMG box transcription factor, characterized by the presence of a high-mobility group domain (HMG) (Wegner, 1999). SOX6 is expressed in several tissues, including cartilage, testis, neural cells, and erythroblasts (Hagiwara, 2011). Mice with a chromosomal inversion (p^100*H*^) disrupting the *Sox6* gene, or carrying a targeted inactivation of Sox6 die perinatally, secondary to cardiac or skeletal myopathy (Hagiwara et al., 2000). *Sox6*-null mouse fetuses and pups are anemic and have defective RBCs (Dumitriu et al., 2006). In erythroid cells, SOX6 has indeed a dual role: it stimulates erythroid cell survival, proliferation, and terminal maturation during definitive murine erythropoiesis (Cantu et al., 2011), and it directly silences embryo/fetal globin genes (Yi et al., 2006; Xu et al., 2010). The first aspect is mediated by the activation of SOCS3, whose overexpression recapitulates the proliferation arrest imposed by SOX6 (Cantu et al., 2011); the second requires the direct binding and repression of the embryonic εy-globin promoter and the cooperation with BCL11A, via direct physical interaction, to silence γ-globin in adult erythroid cells (Yi et al., 2006; Xu et al., 2010).

The DRED complex (direct repeat erythroid-definitive) is a 540-kDa complex containing the nuclear orphan receptors TR2 and TR4, expressed in many tissues, including erythroid cells (Tanabe et al., 2002; Lin et al., 2017). TR2 and TR4 form homodimers or heterodimers binding to GGTCA repeat sequences with variable spacing, a consensus common to non-steroid nuclear receptors (Lee et al., 1998). The double conditional knockout of TR2 and TR4 in mouse erythroid cells results in increased embryonic εy and βh1 globins (Cui et al., 2015). In line with this result, the −117HPFH point mutation, associated with high HbF, reduces TR2/TR4 binding (Tanabe et al., 2002). Finally, TR2 and TR4 have been proposed directly repress GATA1 transcription, suggesting a wider role in erythroid maturation (Tanabe et al., 2007b).

ZBTB7A/LRF is a C2H2 zinc finger TF belonging to the POK (BTB/POZ and Krüppel) group of transcriptional regulators (Davies et al., 1999). ZBTB7A is expressed in various hematopoietic lineages (Maeda, 2016). However, its knockout shows a specific erythroid defect, with mouse embryos dying around E16.5 because of severe anemia, demonstrating that ZBTB7A is required for definitive erythropoiesis (Maeda et al., 2009). Adult-stage knockout of Zbtb7a results in erythropoietin-unresponsive macrocytic anemia, reversed by BIM knockout (Maeda et al., 2009). ZBTB7A specifically represses embryonic and fetal globin gene expression, independently from BCL11A, probably through the interaction with components of the nucleosome remodeling deacetylase (NuRD) complex (Masuda et al., 2016).

## COUP-TFII, the Selective Activator of γ-Globin in Yolk Sac-Derived Cells

The COUP-TFII gene (Chicken Ovalbumin Upstream Promoter Transcriptional Factor II, also known as NR2F2/ARP1) encodes for an orphan nuclear receptor. Its expression is high in the mesenchymal component of developing organs and overall decreases after the completion of organogenesis (Pereira et al., 1995; Lin et al., 2011). Its knockout results in early embryonic lethality (E9.5–E10) (Pereira et al., 1999) caused by defects in angiogenesis and heart development. In the erythroid lineage, Coup-TFII is expressed in the early embryo in the YS and in FL, and it declines around day E12.5 (Filipe et al., 1999; Cui et al., 2015; Fugazza et al., 2020). Although originally identified as ε- and γ-globin gene repressor on the basis of *in vitro* binding data, its functional role remained elusive (Ronchi et al., 1995; Liberati et al., 2001). Our group recently demonstrated that COUP-TFII is co-expressed with embryonic globin genes in cells of YS origin, where it acts as specific γ-globin activator by binding to the GGTCA motifs present within the β-locus (Fugazza et al., 2020). This last observation opens many questions since the same consensus is bound by BCL11A (Liu et al., 2018) and possibly by TR2/TR4 (Tanabe et al., 2007a) in adult cells. Importantly, COUP-TFII is able to activate γ-globin when overexpressed in adult cells, suggesting once again a very similar cellular environment of fetal vs. adult cells (Fugazza et al., 2020).

## Inappropriate Timing of γ-Globin Expression in “Adult-Type” Cells: Hereditary Persistence of Fetal Hemoglobin

HPFH is a benign condition in which γ-globin remains expressed at high levels in adult life (Forget, 1998). HbF can be high in all red blood cells (pancellular HPFH) or restricted to a small subset of erythroid cells (heterocellular HPFH) (Thein et al., 2009). HPFHs, based on the different types of causative mutations, can be broadly divided in three main categories: deletional HPFH, non-deletional HPFH, and HPFH non-linked with the β-locus. Deletional HPFH is associated with the deletion of large regions of DNA between the γ- and β-globin genes within the β-globin locus, as it happens, for example, in the Sicilian ≈13 kb and in the Italian ≈40-kb deletions^1^ (Kountouris et al., 2014). Many deletions include the loss of δ- and β-globin genes resulting in (δβ)^0^-thalessemia and HPFH (Ottolenghi et al., 1982). The molecular mechanism underlying the elevated HbF is complex and involves the concomitant deletion of the β promoter, which removes its competition with the γ promoters for the upstream LCR and for limiting TFs (Forget, 1998). Non-deletional HPFHs are caused by point mutations described in both γ-globin promoters. These mutations fall into three distinct clusters: the −200 region, the −175 site, and the distal CCAAT box region, around −115 (Forget, 1998; Martyn et al., 2018), where they either disrupt binding sites for γ-globin repressors or create *de novo* binding sites for γ-globin activators. For example, the −196 mutation abolishes the binding of ZBTB7A (Martyn et al., 2018), whereas mutations in the distal CCAAT box region impair BCL11A binding (Liu et al., 2018; Martyn et al., 2018). Instead, the −175T > C mutation creates a *de novo* binding site for the activator TAL1 (Wienert et al., 2015). Despite these evidences, the function of these sequences is more complex. The −198T > C mutation, for example, although being located within the cluster of HPFH mutations that impair the binding of ZBTB7A, is a gain-of-function mutation creating a binding site for the erythroid activator KLF1 (Wienert et al., 2017). The same is true for the mutation at position −113 A > G, which, although lying within the −115 region bound by the repressor BCL11A, creates a new binding site for the activator GATA1, without altering the BCL11A binding (Martyn et al., 2019).

## The Role of Modifiers Loci

Genome-wide association studies (GWAS) have revealed other two loci, not linked to the β-locus, consistently associated with HbF levels, and with β-globin disorder severity, across various ethnic backgrounds: a region on 2p (Menzel et al., 2007; Lettre et al., 2008; Uda et al., 2008) and the HBS1L-MYB intergenic region on 6q (Craig et al., 1996; Thein et al., 2007). The 2p region turned out to correspond to BCL11A, and the fine mapping of the single nucleotide polymorphisms associated to HbF within this region led to the identification of the intronic enhancer driving the expression of BCL11A in erythroid cells (Bauer et al., 2013; Canver et al., 2015).

The variants within the HBS1L-MYB intergenic region on 6q impair the binding of LDB1, GATA1, TAL1, and KLF1 to the enhancer controlling *c-MYB* expression (Stadhouders et al., 2014). c-Myb is the cellular homolog of *v-Myb*, the avian retroviral oncogene causing myelomas and lymphomas in birds (Wolff, 1996). Of the two major isoforms, isoform 2 (72 kDa) is the dominant one in human erythroid cells (Baker et al., 2010; Wang et al., 2018). In hematopoiesis, *c-MYB* is expressed in immature cells of all hematopoietic lineages (Wang et al., 2018); in erythropoiesis, it is required for the expansion of erythroid progenitors and must be downregulated to allow differentiation (Emambokus et al., 2003). c-Myb-null murine embryos are normal until E13.5, but by E15, they become severely anemic and die, suggesting that c-Myb is required for definitive erythropoiesis (Mucenski et al., 1991). The reduced c-MYB level has a twofold impact on globin genes. Low c-MYB levels, by accelerating the kinetics of erythroid differentiation, would favor the release of early erythroid progenitor cells still synthetizing HbF (Stamatoyannopoulos, 2005; Jiang et al., 2006). In addition, the reduced activation of KLF1 (Bianchi et al., 2010; Suzuki et al., 2013) by MYB would promote γ-globin expression by reducing BCL11A levels.

## Inappropriate Timing of γ-Globin Expression in “Fetal-Type” Cells Persisting After Birth: The Case of Juvenile Myelomonocytic Leukemia

High HbF levels are a hallmark of different leukemias (Sheridan et al., 1976). However, the expression of γ-globin in juvenile myelomonocytic leukemia (JMML) is peculiar. JMML is a rare and aggressive blood cancer of early childhood (Loh, 2011; Niemeyer and Flotho, 2019). About 90% of the patients present hyperactivation of the RAS pathway, as a result of mutations in KRAS, NRAS, PTPN11, NF1, or CBL genes, and about 25% of the patients carry chromosome 7 monosomy (Flotho et al., 1999; de Vries et al., 2010). JMML is considered a stem cell disease (Inoue et al., 1987; Busque et al., 1995; Flotho et al., 1999; Cooper et al., 2000). Of interest, increased HbF levels and the presence of fetal red cell traits (Weinberg et al., 1990; de Vries et al., 2010; Helsmoortel et al., 2016a) are present in more than half of JMML patients. These evidences suggested a fetal origin for JMML, confirmed by the retrospective analysis of JMML patient samples collected at birth (Kratz et al., 2005; Matsuda et al., 2010; Stieglitz et al., 2015). However, yolk sac EMPs expressing gain of function PTPN11 mutations recapitulate part of the characteristics of JMML, but they are not able to cause disease in mice (Tarnawsky et al., 2017). Recently, gene expression profiling of JMML samples identified a subgroup characterized by high LIN28B expression and higher HbF levels (Helsmoortel et al., 2016b). LIN28B is an oncofetal protein (Shyh-Chang and Daley, 2013) that induces γ-globin expression (Lee et al., 2013, 2015; de Vasconcellos et al., 2014) and is highly expressed in fetal HSCs (Copley et al., 2013). Interestingly, LIN28B was shown to repress BCL11A-XL by blocking its translation (Basak et al., 2020). Thus, high levels of LIN28B decrease the amount of BCL11A protein, and this results in high HbF. This observation suggests a mechanistic link between LIN28B and high HbF observed in JMML. Whether the 50% of JMML with high HbF arose from a fetal EMP subpopulation expressing high LIN28B deserves further investigation.

## Conclusion

The existence of γ-globin genes is specific to humans and old-world monkeys. Physiologically, γ-globin expression is confined to fetal life, tightly regulated by a complex network of transcription factors (activators and repressors) and co-regulators. Nevertheless, normal subjects present rare F cells, whose nature is still unclear (Rochette et al., 1994). During development, γ-globin genes are expressed in two different types of erythroblasts, with independent origin, the first originating from the transient, yolk sac-derived EMP population and the second from definitive HSCs (McGrath et al., 2011). These two cell types are very similar in their maturational pathway, and adult cells indeed represent a permissive environment for γ-globin expression, as shown by HPFH. Here, alterations in the few specific transcription factors that regulate γ-globin transcription (or in the sequences bound by them within the HBB locus) allow substantial γ-globin expression. Moreover, recent RNA-seq studies on A and F cells from the same healthy donors show that these cells do not significantly differ in the expression of known γ-globin regulators, suggesting that differences in the transcription of globin genes within the HBB locus itself account for the HbF trait (Khandros et al., 2020).

However, new evidence from the study of JMML unveil an alternative possible scenario, where γ-globin genes could be the marker of the fetal origin of these leukemic cells. The discovery that fetal progenitor-derived cells can persist to adulthood and contribute to disease raises the possibility that, especially in childhood malignancies, such as JMML, HbF can indeed be the signature of the fetal origin of cancer cells.

## Author’s Note

While this minireview was in press, an article was published by Liu and colleagues (doi: 10.1038/s41588-021-00798-y) showing that BCL11A competes with NF-Y binding to initiate γ-globin repression at the CCAAT box region.

## Author Contributions

AR conceived and wrote the manuscript. GB and AL contributed with ideas and discussion. SS and AA contributed in the organization of the manuscript. All authors contributed to the article and approved the submitted version.

## Conflict of Interest

The authors declare that the research was conducted in the absence of any commercial or financial relationships that could be construed as a potential conflict of interest. The handling editor declared a past co-authorship with one of the authors AR.

## Footnotes

1 https://www.ithanet.eu/

## References

[B1] Aisen P. (2004). Transferrin receptor 1. *Int. J. Biochem. Cell Biol.* 36 2137–2143. 15313461 10.1016/j.biocel.2004.02.007

[B2] Avram D. Fields A. Pretty On Top K. Nevrivy D. J. Ishmael J. E. Leid M. (2000). Isolation of a novel family of C(2)H(2) zinc finger proteins implicated in transcriptional repression mediated by chicken ovalbumin upstream promoter transcription factor (COUP-TF) orphan nuclear receptors. *J. Biol. Chem.* 275 10315–10322. 10.1074/jbc.275.14.10315 10744719 PMC2819356

[B3] Baker S. J. Kumar A. Reddy E. P. (2010). p89c-Myb is not required for fetal or adult hematopoiesis. *Genesis* 48 309–316. 20196078 10.1002/dvg.20619 PMC2868939

[B4] Barbarani G. Fugazza C. Strouboulis J. Ronchi A. E. (2019). The pleiotropic effects of GATA1 and KLF1 in physiological erythropoiesis and in dyserythropoietic disorders. *Front. Physiol.* 10:91. 10.3389/fphys.2019.00091 30809156 PMC6379452

[B5] Baron M. H. (2013). Concise review: early embryonic erythropoiesis: not so primitive after all. *Stem Cells* 31 849–856. 10.1002/stem.1342 23361843 PMC3637392

[B6] Basak A. Hancarova M. Ulirsch J. C. Balci T. B. Trkova M. Pelisek M. (2015). BCL11A deletions result in fetal hemoglobin persistence and neurodevelopmental alterations. *J. Clin. Invest.* 125 2363–2368. 10.1172/jci81163 25938782 PMC4497765

[B7] Basak A. Munschauer M. Lareau C. A. Montbleau K. E. Ulirsch J. C. Hartigan C. R. (2020). Control of human hemoglobin switching by LIN28B-mediated regulation of BCL11A translation. *Nat. Genet.* 52 138–145. 10.1038/s41588-019-0568-7 31959994 PMC7031047

[B8] Bauer D. E. Kamran S. C. Lessard S. Xu J. Fujiwara Y. Lin C. (2013). An erythroid enhancer of BCL11A subject to genetic variation determines fetal hemoglobin level. *Science* 342 253–257. 10.1126/science.1242088 24115442 PMC4018826

[B9] Bianchi E. Zini R. Salati S. Tenedini E. Norfo R. Tagliafico E. (2010). c-myb supports erythropoiesis through the transactivation of KLF1 and LMO2 expression. *Blood* 116 e99–e110. 20686118 10.1182/blood-2009-08-238311

[B10] Borg J. Papadopoulos P. Georgitsi M. Gutierrez L. Grech G. Fanis P. (2010). Haploinsufficiency for the erythroid transcription factor KLF1 causes hereditary persistence of fetal hemoglobin. *Nat. Genet.* 42 801–805. 10.1038/ng.630 20676099 PMC2930131

[B11] Boyer S. H. Belding T. K. Margolet L. Noyes A. N. (1975). Fetal hemoglobin restriction to a few erythrocytes (F cells) in normal human adults. *Science* 188 361–363. 10.1126/science.804182 804182

[B12] Busque L. Gilliland D. G. Prchal J. T. Sieff C. A. Weinstein H. J. Sokol J. M. (1995). Clonality in juvenile chronic myelogenous leukemia. *Blood* 85 21–30. 10.1182/blood.v85.1.21.bloodjournal85121 7803795

[B13] Cantu C. Ierardi R. Alborelli I. Fugazza C. Cassinelli L. Piconese S. (2011). Sox6 enhances erythroid differentiation in human erythroid progenitors. *Blood* 117 3669–3679. 10.1182/blood-2010-04-282350 21263153

[B14] Canver M. C. Smith E. C. Sher F. Pinello L. Sanjana N. E. Shalem O. (2015). BCL11A enhancer dissection by Cas9-mediated in situ saturating mutagenesis. *Nature* 527 192–197. 10.1038/nature15521 26375006 PMC4644101

[B15] Chen K. Liu J. Heck S. Chasis J. A. An X. Mohandas N. (2009). Resolving the distinct stages in erythroid differentiation based on dynamic changes in membrane protein expression during erythropoiesis. *Proc. Natl. Acad. Sci. U.S.A.* 106 17413–17418. 10.1073/pnas.0909296106 19805084 PMC2762680

[B16] Cooper L. J. Shannon K. M. Loken M. R. Weaver M. Stephens K. Sievers E. L. (2000). Evidence that juvenile myelomonocytic leukemia can arise from a pluripotential stem cell. *Blood* 96 2310–2313. 10.1182/blood.v96.6.2310.h8002310_2310_2313 10979983

[B17] Copley M. R. Babovic S. Benz C. Knapp D. J. Beer P. A. Kent D. G. (2013). The Lin28b-let-7-Hmga2 axis determines the higher self-renewal potential of fetal haematopoietic stem cells. *Nat. Cell Biol.* 15 916–925. 10.1038/ncb2783 23811688

[B18] Craig J. E. Rochette J. Fisher C. A. Weatherall D. J. Marc S. Lathrop G. M. (1996). Dissecting the loci controlling fetal haemoglobin production on chromosomes 11p and 6q by the regressive approach. *Nat. Genet.* 12 58–64. 10.1038/ng0196-58 8528252

[B19] Cui S. Tanabe O. Sierant M. Shi L. Campbell A. Lim K. C. (2015). Compound loss of function of nuclear receptors Tr2 and Tr4 leads to induction of murine embryonic beta-type globin genes. *Blood* 125 1477–1487. 10.1182/blood-2014-10-605022 25561507 PMC4342359

[B20] Davies J. M. Hawe N. Kabarowski J. Huang Q. H. Zhu J. Brand N. J. (1999). Novel BTB/POZ domain zinc-finger protein, LRF, is a potential target of the LAZ-3/BCL-6 oncogene. *Oncogene* 18 365–375. 10.1038/sj.onc.1202332 9927193

[B21] de Vasconcellos J. F. Fasano R. M. Lee Y. T. Kaushal M. Byrnes C. Meier E. R. (2014). LIN28A expression reduces sickling of cultured human erythrocytes. *PLoS One* 9:e106924. 10.1371/journal.pone.0106924 25188417 PMC4154803

[B22] de Vries A. C. Zwaan C. M. van den Heuvel-Eibrink M. M. (2010). Molecular basis of juvenile myelomonocytic leukemia. *Haematologica* 95 179–182. 10.3324/haematol.2009.016865 20139388 PMC2817017

[B23] Dumitriu B. Patrick M. R. Petschek J. P. Cherukuri S. Klingmuller U. Fox P. L. (2006). Sox6 cell-autonomously stimulates erythroid cell survival, proliferation, and terminal maturation and is thereby an important enhancer of definitive erythropoiesis during mouse development. *Blood* 108 1198–1207. 10.1182/blood-2006-02-004184 16627753

[B24] Dzierzak E. Bigas A. (2018). Blood development: hematopoietic stem cell dependence and independence. *Cell Stem Cell* 22 639–651. 10.1016/j.stem.2018.04.015 29727679

[B25] Dzierzak E. Philipsen S. (2013). Erythropoiesis: development and differentiation. *Cold Spring Harb. Perspect. Med.* 3:a011601. 10.1101/cshperspect.a011601 23545573 PMC3684002

[B26] Emambokus N. Vegiopoulos A. Harman B. Jenkinson E. Anderson G. Frampton J. (2003). Progression through key stages of haemopoiesis is dependent on distinct threshold levels of c-Myb. *EMBO J.* 22 4478–4488. 10.1093/emboj/cdg434 12941699 PMC202376

[B27] Epelman S. Lavine K. J. Beaudin A. E. Sojka D. K. Carrero J. A. Calderon B. (2014). Embryonic and adult-derived resident cardiac macrophages are maintained through distinct mechanisms at steady state and during inflammation. *Immunity* 40 91–104. 10.1016/j.immuni.2013.11.019 24439267 PMC3923301

[B28] Feng W. C. Southwood C. M. Bieker J. J. (1994). Analyses of beta-thalassemia mutant DNA interactions with erythroid Kruppel-like factor (EKLF), an erythroid cell-specific transcription factor. *J. Biol. Chem.* 269 1493–1500. 10.1016/s0021-9258(17)42283-6 8288615

[B29] Ferreira R. Ohneda K. Yamamoto M. Philipsen S. (2005). GATA1 function, a paradigm for transcription factors in hematopoiesis. *Mol. Cell Biol.* 25 1215–1227. 10.1128/mcb.25.4.1215-1227.2005 15684376 PMC548021

[B30] Filipe A. Li Q. Deveaux S. Godin I. Romeo P. H. Stamatoyannopoulos G. (1999). Regulation of embryonic/fetal globin genes by nuclear hormone receptors: a novel perspective on hemoglobin switching. *EMBO J.* 18 687–697. 10.1093/emboj/18.3.687 9927428 PMC1171161

[B31] Flotho C. Valcamonica S. Mach-Pascual S. Schmahl G. Corral L. Ritterbach J. (1999). RAS mutations and clonality analysis in children with juvenile myelomonocytic leukemia (JMML). *Leukemia* 13 32–37. 10.1038/sj.leu.2401240 10049057

[B32] Forget B. G. (1990). Developmental control of human globin gene expression. *Prog. Clin. Biol. Res.* 352 313–322. 1698299

[B33] Forget B. G. (1998). Molecular basis of hereditary persistence of fetal hemoglobin. *Ann. N. Y. Acad. Sci.* 850 38–44. 10.1111/j.1749-6632.1998.tb10460.x 9668525

[B34] Fugazza C. Barbarani G. Elangovan S. Marini M. G. Giolitto S. Font-Monclus I. (2020). The Coup-TFII orphan nuclear receptor is an activator of the gamma-globin gene. *Haematologica* 106 474–482. 10.3324/haematol.2019.241224 32107331 PMC7849756

[B35] Funnell A. P. Prontera P. Ottaviani V. Piccione M. Giambona A. Maggio A. (2015). 2p15-p16.1 microdeletions encompassing and proximal to BCL11A are associated with elevated HbF in addition to neurologic impairment. *Blood* 126 89–93. 10.1182/blood-2015-04-638528 26019277 PMC4492199

[B36] Gasiorek J. J. Blank V. (2015). Regulation and function of the NFE2 transcription factor in hematopoietic and non-hematopoietic cells. *Cell. Mol. Life Sci.* 72 2323–2335. 10.1007/s00018-015-1866-6 25721735 PMC11114048

[B37] Gomez Perdiguero E. Klapproth K. Schulz C. Busch K. Azzoni E. Crozet L. (2015). Tissue-resident macrophages originate from yolk-sac-derived erythro-myeloid progenitors. *Nature* 518 547–551. 10.1038/nature13989 25470051 PMC5997177

[B38] Greig L. C. Woodworth M. B. Greppi C. Macklis J. D. (2016). Ctip1 controls acquisition of sensory area identity and establishment of sensory input fields in the developing neocortex. *Neuron* 90 261–277. 10.1016/j.neuron.2016.03.008 27100196 PMC4873772

[B39] Grosveld F. van Assendelft G. B. Greaves D. R. Kollias G. (1987). Position-independent, high-level expression of the human beta-globin gene in transgenic mice. *Cell* 51 975–985. 10.1016/0092-8674(87)90584-8 3690667

[B40] Hagiwara N. (2011). Sox6, jack of all trades: a versatile regulatory protein in vertebrate development. *Dev. Dyn.* 240 1311–1321. 10.1002/dvdy.22639 21495113 PMC3092843

[B41] Hagiwara N. Klewer S. E. Samson R. A. Erickson D. T. Lyon M. F. Brilliant M. H. (2000). Sox6 is a candidate gene for p100H myopathy, heart block, and sudden neonatal death. *Proc. Natl. Acad. Sci. U.S.A.* 97 4180–4185. 10.1073/pnas.97.8.4180 10760285 PMC18189

[B42] Helsmoortel H. H. Bresolin S. Lammens T. Cave H. Noellke P. Caye A. (2016a). LIN28B overexpression defines a novel fetal-like subgroup of juvenile myelomonocytic leukemia. *Blood* 127 1163–1172. 10.1182/blood-2015-09-667808 26712910

[B43] Helsmoortel H. H. De Moerloose B. Pieters T. Ghazavi F. Bresolin S. Cave H. (2016b). LIN28B is over-expressed in specific subtypes of pediatric leukemia and regulates lncRNA H19. *Haematologica* 101 e240–e244. 26969084 10.3324/haematol.2016.143818 PMC5013963

[B44] Higgs D. R. Wood W. G. Jarman A. P. Sharpe J. Lida J. Pretorius I. M. (1990). A major positive regulatory region located far upstream of the human alpha-globin gene locus. *Genes Dev.* 4 1588–1601. 10.1101/gad.4.9.1588 2253879

[B45] Inoue S. Shibata T. Ravindranath Y. Gohle N. (1987). Clonal origin of erythroid cells in juvenile chronic myelogenous leukemia. *Blood* 69 975–976. 10.1182/blood.v69.3.975.bloodjournal693975 3469003

[B46] Ippolito G. C. Dekker J. D. Wang Y. H. Lee B. K. Shaffer A. L. III Lin J. (2014). Dendritic cell fate is determined by BCL11A. *Proc. Natl. Acad. Sci. U.S.A.* 111 E998–E1006. 24591644 10.1073/pnas.1319228111 PMC3964079

[B47] Jiang J. Best S. Menzel S. Silver N. Lai M. I. Surdulescu G. L. (2006). cMYB is involved in the regulation of fetal hemoglobin production in adults. *Blood* 108 1077–1083. 10.1182/blood-2006-01-008912 16861354

[B48] Kang Y. Kim Y. W. Yun J. Shin J. Kim A. (2015). KLF1 stabilizes GATA-1 and TAL1 occupancy in the human beta-globin locus. *Biochim. Biophys. Acta* 1849 282–289. 10.1016/j.bbagrm.2014.12.010 25528728

[B49] Katsumura K. R. Bresnick E. H. Group G. F. M. (2017). The GATA factor revolution in hematology. *Blood* 129 2092–2102. 10.1182/blood-2016-09-687871 28179282 PMC5391619

[B50] Khaled W. T. Choon Lee S. Stingl J. Chen X. Raza Ali H. Rueda O. M. (2015). BCL11A is a triple-negative breast cancer gene with critical functions in stem and progenitor cells. *Nat. Commun.* 6:5987. 10.1038/ncomms6987 PMC4338552 25574598

[B51] Khandros E. Huang P. Peslak S. A. Sharma M. Abdulmalik O. Giardine B. M. (2020). Understanding heterogeneity of fetal hemoglobin induction through comparative analysis of F and A erythroblasts. *Blood* 135 1957–1968. 10.1182/blood.2020005058 32268371 PMC7256358

[B52] Kim Y. W. Yun W. J. Kim A. (2016). Erythroid activator NF-E2, TAL1 and KLF1 play roles in forming the LCR HSs in the human adult beta-globin locus. *Int. J. Biochem. Cell Biol.* 75 45–52. 10.1016/j.biocel.2016.03.013 27026582

[B53] Kina T. Ikuta K. Takayama E. Wada K. Majumdar A. S. Weissman I. L. (2000). The monoclonal antibody TER-119 recognizes a molecule associated with glycophorin A and specifically marks the late stages of murine erythroid lineage. *Br. J. Haematol.* 109 280–287. 10.1046/j.1365-2141.2000.02037.x 10848813

[B54] Kountouris P. Lederer C. W. Fanis P. Feleki X. Old J. Kleanthous M. (2014). IthaGenes: an interactive database for haemoglobin variations and epidemiology. *PLoS One* 9:e103020. 10.1371/journal.pone.0103020 25058394 PMC4109966

[B55] Kratz C. P. Niemeyer C. M. Castleberry R. P. Cetin M. Bergstrasser E. Emanuel P. D. (2005). The mutational spectrum of PTPN11 in juvenile myelomonocytic leukemia and Noonan syndrome/myeloproliferative disease. *Blood* 106 2183–2185. 10.1182/blood-2005-02-0531 15928039 PMC1895140

[B56] Lacaud G. Kouskoff V. (2017). Hemangioblast, hemogenic endothelium, and primitive versus definitive hematopoiesis. *Exp. Hematol.* 49 19–24. 10.1016/j.exphem.2016.12.009 28043822

[B57] Lee C. H. Chinpaisal C. Wei L. N. (1998). A novel nuclear receptor heterodimerization pathway mediated by orphan receptors TR2 and TR4. *J. Biol. Chem.* 273 25209–25215. 10.1074/jbc.273.39.25209 9737983

[B58] Lee Y. T. de Vasconcellos J. F. Byrnes C. Kaushal M. Rabel A. Tumburu L. (2015). Erythroid-specific expression of LIN28A is sufficient for robust gamma-globin gene and protein expression in adult erythroblasts. *PLoS One* 10:e0144977. 10.1371/journal.pone.0144977 26675483 PMC4684222

[B59] Lee Y. T. de Vasconcellos J. F. Yuan J. Byrnes C. Noh S. J. Meier E. R. (2013). LIN28B-mediated expression of fetal hemoglobin and production of fetal-like erythrocytes from adult human erythroblasts ex vivo. *Blood* 122 1034–1041. 10.1182/blood-2012-12-472308 23798711 PMC3739030

[B60] Lettre G. Sankaran V. G. Bezerra M. A. Araujo A. S. Uda M. Sanna S. (2008). DNA polymorphisms at the BCL11A, HBS1L-MYB, and beta-globin loci associate with fetal hemoglobin levels and pain crises in sickle cell disease. *Proc. Natl. Acad. Sci. U.S.A.* 105 11869–11874. 10.1073/pnas.0804799105 18667698 PMC2491485

[B61] Liberati C. Cera M. R. Secco P. Santoro C. Mantovani R. Ottolenghi S. (2001). Cooperation and competition between the binding of COUP-TFII and NF-Y on human epsilon- and gamma-globin gene promoters. *J. Biol. Chem.* 276 41700–41709. 10.1074/jbc.m102987200 11544252

[B62] Liberati C. Ronchi A. Lievens P. Ottolenghi S. Mantovani R. (1998). NF-Y organizes the gamma-globin CCAAT boxes region. *J. Biol. Chem.* 273 16880–16889. 10.1074/jbc.273.27.16880 9642249

[B63] Lin F. J. Qin J. Tang K. Tsai S. Y. Tsai M. J. (2011). Coup d’Etat: an orphan takes control. *Endocr. Rev.* 32 404–421. 10.1210/er.2010-0021 21257780 PMC3365794

[B64] Lin S. J. Yang D. R. Yang G. Lin C. Y. Chang H. C. Li G. (2017). TR2 and TR4 orphan nuclear receptors: an overview. *Curr. Top. Dev. Biol.* 125 357–373. 10.1016/bs.ctdb.2017.02.002 28527578

[B65] Liu H. Ippolito G. C. Wall J. K. Niu T. Probst L. Lee B. S. (2006). Functional studies of BCL11A: characterization of the conserved BCL11A-XL splice variant and its interaction with BCL6 in nuclear paraspeckles of germinal center B cells. *Mol. Cancer* 5:18. 10.1186/1476-4598-5-18 PMC1526750 16704730

[B66] Liu N. Hargreaves V. V. Zhu Q. Kurland J. V. Hong J. Kim W. (2018). Direct promoter repression by BCL11A controls the fetal to adult hemoglobin switch. *Cell* 173 430–442.e17. 29606353 10.1016/j.cell.2018.03.016 PMC5889339

[B67] Liu P. Keller J. R. Ortiz M. Tessarollo L. Rachel R. A. Nakamura T. (2003). Bcl11a is essential for normal lymphoid development. *Nat. Immunol.* 4 525–532. 10.1038/ni925 12717432

[B68] Loh M. L. (2011). Recent advances in the pathogenesis and treatment of juvenile myelomonocytic leukaemia. *Br. J. Haematol.* 152 677–687. 10.1111/j.1365-2141.2010.08525.x 21623760

[B69] Love P. E. Warzecha C. Li L. (2014). Ldb1 complexes: the new master regulators of erythroid gene transcription. *Trends Genet.* 30 1–9. 10.1016/j.tig.2013.10.001 24290192 PMC3882320

[B70] Luc S. Huang J. McEldoon J. L. Somuncular E. Li D. Rhodes C. (2016). Bcl11a deficiency leads to hematopoietic stem cell defects with an aging-like phenotype. *Cell Rep.* 16 3181–3194. 10.1016/j.celrep.2016.08.064 27653684 PMC5054719

[B71] Maeda T. (2016). Regulation of hematopoietic development by ZBTB transcription factors. *Int. J. Hematol.* 104 310–323. 10.1007/s12185-016-2035-x 27250345 PMC5001899

[B72] Maeda T. Ito K. Merghoub T. Poliseno L. Hobbs R. M. Wang G. (2009). LRF is an essential downstream target of GATA1 in erythroid development and regulates BIM-dependent apoptosis. *Dev. Cell* 17 527–540. 10.1016/j.devcel.2009.09.005 19853566 PMC3134301

[B73] Martyn G. E. Quinlan K. G. R. Crossley M. (2017). The regulation of human globin promoters by CCAAT box elements and the recruitment of NF-Y. *Biochim. Biophys. Acta Gene Regul. Mech.* 1860 525–536. 10.1016/j.bbagrm.2016.10.002 27718361

[B74] Martyn G. E. Wienert B. Kurita R. Nakamura Y. Quinlan K. G. R. Crossley M. (2019). A natural regulatory mutation in the proximal promoter elevates fetal globin expression by creating a de novo GATA1 site. *Blood* 133 852–856. 10.1182/blood-2018-07-863951 30617196

[B75] Martyn G. E. Wienert B. Yang L. Shah M. Norton L. J. Burdach J. (2018). Natural regulatory mutations elevate the fetal globin gene via disruption of BCL11A or ZBTB7A binding. *Nat. Genet.* 50 498–503. 10.1038/s41588-018-0085-0 29610478

[B76] Masuda T. Wang X. Maeda M. Canver M. C. Sher F. Funnell A. P. (2016). Transcription factors LRF and BCL11A independently repress expression of fetal hemoglobin. *Science* 351 285–289. 10.1126/science.aad3312 26816381 PMC4778394

[B77] Matsuda K. Sakashita K. Taira C. Tanaka-Yanagisawa M. Yanagisawa R. Shiohara M. (2010). Quantitative assessment of PTPN11 or RAS mutations at the neonatal period and during the clinical course in patients with juvenile myelomonocytic leukaemia. *Br. J. Haematol.* 148 593–599. 10.1111/j.1365-2141.2009.07968.x 19874312

[B78] McGrath K. E. Frame J. M. Fromm G. J. Koniski A. D. Kingsley P. D. Little J. (2011). A transient definitive erythroid lineage with unique regulation of the beta-globin locus in the mammalian embryo. *Blood* 117 4600–4608. 10.1182/blood-2010-12-325357 21378272 PMC3099576

[B79] Menzel S. Garner C. Gut I. Matsuda F. Yamaguchi M. Heath S. (2007). A QTL influencing F cell production maps to a gene encoding a zinc-finger protein on chromosome 2p15. *Nat. Genet.* 39 1197–1199. 10.1038/ng2108 17767159

[B80] Miller I. J. Bieker J. J. (1993). A novel, erythroid cell-specific murine transcription factor that binds to the CACCC element and is related to the Kruppel family of nuclear proteins. *Mol. Cell Biol.* 13 2776–2786. 10.1128/mcb.13.5.2776 7682653 PMC359658

[B81] Mucenski M. L. Mclain K. Kier A. B. Swerdlow S. H. Schreiner C. M. Miller T. A. (1991). A Functional C-Myb gene is required for normal murine fetal hepatic hematopoiesis. *Cell* 65 677–689. 10.1016/0092-8674(91)90099-k 1709592

[B82] Niemeyer C. M. Flotho C. (2019). Juvenile myelomonocytic leukemia: who’s the driver at the wheel? *Blood* 133 1060–1070. 10.1182/blood-2018-11-844688 30670449

[B83] Nuez B. Michalovich D. Bygrave A. Ploemacher R. Grosveld F. (1995). Defective haematopoiesis in fetal liver resulting from inactivation of the EKLF gene. *Nature* 375 316–318. 10.1038/375316a0 7753194

[B84] Orkin S. H. Zon L. I. (2008). Hematopoiesis: an evolving paradigm for stem cell biology. *Cell* 132 631–644. 10.1016/j.cell.2008.01.025 18295580 PMC2628169

[B85] Ottolenghi S. Giglioni B. Taramelli R. Comi P. Gianni A. M. (1982). delta beta-Thalassemia and HPFH. *Birth Defects Orig. Artic. Ser.* 18 65–67. 6186314

[B86] Palis J. (2014). Primitive and definitive erythropoiesis in mammals. *Front. Physiol.* 5:3. 10.3389/fphys.2014.00003 24478716 PMC3904103

[B87] Palstra R. J. Tolhuis B. Splinter E. Nijmeijer R. Grosveld F. de Laat W. (2003). The beta-globin nuclear compartment in development and erythroid differentiation. *Nat. Genet.* 35 190–194. 10.1038/ng1244 14517543

[B88] Peiris H. Park S. Louis S. Gu X. Lam J. Y. Asplund O. (2018). Discovering human diabetes-risk gene function with genetics and physiological assays. *Nat. Commun.* 9:3855. 10.1038/s41467-018-06249-3 PMC6155000 30242153

[B89] Pereira F. A. Qiu Y. Tsai M. J. Tsai S. Y. (1995). Chicken ovalbumin upstream promoter transcription factor (COUP-TF): expression during mouse embryogenesis. *J. Steroid Biochem. Mol. Biol.* 53 503–508. 10.1016/0960-0760(95)00097-j 7626501

[B90] Pereira F. A. Qiu Y. Zhou G. Tsai M. J. Tsai S. Y. (1999). The orphan nuclear receptor COUP-TFII is required for angiogenesis and heart development. *Genes Dev.* 13 1037–1049. 10.1101/gad.13.8.1037 10215630 PMC316637

[B91] Perkins A. Xu X. Higgs D. R. Patrinos G. P. Arnaud L. Bieker J. J. (2016). Kruppeling erythropoiesis: an unexpected broad spectrum of human red blood cell disorders due to KLF1 variants. *Blood* 127 1856–1862. 10.1182/blood-2016-01-694331 26903544 PMC4832505

[B92] Perkins A. C. Sharpe A. H. Orkin S. H. (1995). Lethal beta-thalassaemia in mice lacking the erythroid CACCC-transcription factor EKLF. *Nature* 375 318–322. 10.1038/375318a0 7753195

[B93] Peterson K. R. Li Q. L. Clegg C. H. Furukawa T. Navas P. A. Norton E. J. (1995). Use of yeast artificial chromosomes (YACs) in studies of mammalian development: production of beta-globin locus YAC mice carrying human globin developmental mutants. *Proc. Natl. Acad. Sci. U.S.A.* 92 5655–5659. 10.1073/pnas.92.12.5655 7539923 PMC41755

[B94] Philipsen S. Hardison R. C. (2018). Evolution of hemoglobin loci and their regulatory elements. *Blood Cells Mol. Dis.* 70 2–12. 10.1016/j.bcmd.2017.08.001 28811072 PMC5807248

[B95] Piskounova E. Polytarchou C. Thornton J. E. LaPierre R. J. Pothoulakis C. Hagan J. P. (2011). Lin28A and Lin28B inhibit let-7 microRNA biogenesis by distinct mechanisms. *Cell* 147 1066–1079. 10.1016/j.cell.2011.10.039 22118463 PMC3227872

[B96] Rochette J. Craig J. E. Thein S. L. (1994). Fetal hemoglobin levels in adults. *Blood Rev.* 8 213–224. 10.1016/0268-960x(94)90109-0 7534152

[B97] Ronchi A. E. Bottardi S. Mazzucchelli C. Ottolenghi S. Santoro C. (1995). Differential binding of the NFE3 and CP1/NFY transcription factors to the human gamma- and epsilon-globin CCAAT boxes. *J. Biol. Chem.* 270 21934–21941. 10.1074/jbc.270.37.21934 7545172

[B98] Sankaran V. G. Menne T. F. Xu J. Akie T. E. Lettre G. Van Handel B. (2008). Human fetal hemoglobin expression is regulated by the developmental stage-specific repressor BCL11A. *Science* 322 1839–1842. 10.1126/science.1165409 19056937

[B99] Sankaran V. G. Xu J. Orkin S. H. (2010a). Advances in the understanding of haemoglobin switching. *Br. J. Haematol.* 149 181–194. 10.1111/j.1365-2141.2010.08105.x 20201948 PMC4153468

[B100] Sankaran V. G. Xu J. Orkin S. H. (2010b). Transcriptional silencing of fetal hemoglobin by BCL11A. *Ann. N. Y. Acad. Sci.* 1202 64–68. 10.1111/j.1749-6632.2010.05574.x 20712774

[B101] Sankaran V. G. Xu J. Ragoczy T. Ippolito G. C. Walkley C. R. Maika S. D. (2009). Developmental and species-divergent globin switching are driven by BCL11A. *Nature* 460 1093–1097. 10.1038/nature08243 19657335 PMC3749913

[B102] Sheridan B. L. Weatherall D. J. Clegg J. B. Pritchard J. Wood W. G. Callender S. T. (1976). The patterns of fetal haemoglobin production in leukaemia. *Br. J. Haematol.* 32 487–506. 10.1111/j.1365-2141.1976.tb00952.x 816370

[B103] Shyh-Chang N. Daley G. Q. (2013). Lin28: primal regulator of growth and metabolism in stem cells. *Cell Stem Cell* 12 395–406. 10.1016/j.stem.2013.03.005 23561442 PMC3652335

[B104] Shyh-Chang N. Zhu H. Yvanka de Soysa T. Shinoda G. Seligson M. T. Tsanov K. M. (2013). Lin28 enhances tissue repair by reprogramming cellular metabolism. *Cell* 155 778–792. 10.1016/j.cell.2013.09.059 24209617 PMC3917449

[B105] Stadhouders R. Aktuna S. Thongjuea S. Aghajanirefah A. Pourfarzad F. van Ijcken W. (2014). HBS1L-MYB intergenic variants modulate fetal hemoglobin via long-range MYB enhancers. *J. Clin. Invest.* 124 1699–1710. 10.1172/jci71520 24614105 PMC3973089

[B106] Stamatoyannopoulos G. (1991). Human hemoglobin switching. *Science* 252:383. 10.1126/science.2017679 2017679

[B107] Stamatoyannopoulos G. (2005). Control of globin gene expression during development and erythroid differentiation. *Exp. Hematol.* 33 259–271. 10.1016/j.exphem.2004.11.007 15730849 PMC2819985

[B108] Stieglitz E. Taylor-Weiner A. N. Chang T. Y. Gelston L. C. Wang Y. D. Mazor T. (2015). The genomic landscape of juvenile myelomonocytic leukemia. *Nat. Genet.* 47 1326–1333. 26457647 10.1038/ng.3400 PMC4626387

[B109] Strouboulis J. Dillon N. Grosveld F. (1992). Developmental regulation of a complete 70-kb human beta-globin locus in transgenic mice. *Genes Dev.* 6 1857–1864. 10.1101/gad.6.10.1857 1383089

[B110] Suzuki M. Yamazaki H. Mukai H. Y. Motohashi H. Shi L. Tanabe O. (2013). Disruption of the Hbs1l-Myb locus causes hereditary persistence of fetal hemoglobin in a mouse model. *Mol. Cell Biol.* 33 1687–1695. 10.1128/mcb.01617-12 23428869 PMC3624254

[B111] Tanabe O. Katsuoka F. Campbell A. D. Song W. Yamamoto M. Tanimoto K. (2002). An embryonic/fetal beta-type globin gene repressor contains a nuclear receptor TR2/TR4 heterodimer. *EMBO J.* 21 3434–3442. 10.1093/emboj/cdf340 12093744 PMC126089

[B112] Tanabe O. McPhee D. Kobayashi S. Shen Y. Brandt W. Jiang X. (2007a). Embryonic and fetal beta-globin gene repression by the orphan nuclear receptors, TR2 and TR4. *EMBO J.* 26 2295–2306. 10.1038/sj.emboj.7601676 17431400 PMC1864974

[B113] Tanabe O. Shen Y. Liu Q. Campbell A. D. Kuroha T. Yamamoto M. (2007b). The TR2 and TR4 orphan nuclear receptors repress Gata1 transcription. *Genes Dev.* 21 2832–2844. 10.1101/gad.1593307 17974920 PMC2045135

[B114] Tarnawsky S. P. Yoshimoto M. Deng L. Chan R. J. Yoder M. C. (2017). Yolk sac erythromyeloid progenitors expressing gain of function PTPN11 have functional features of JMML but are not sufficient to cause disease in mice. *Dev. Dyn.* 246 1001–1014. 10.1002/dvdy.24598 28975680 PMC5828034

[B115] Thein S. L. Menzel S. Lathrop M. Garner C. (2009). Control of fetal hemoglobin: new insights emerging from genomics and clinical implications. *Hum. Mol. Genet.* 18 R216–R223. 19808799 10.1093/hmg/ddp401 PMC2758709

[B116] Thein S. L. Menzel S. Peng X. Best S. Jiang J. Close J. (2007). Intergenic variants of HBS1L-MYB are responsible for a major quantitative trait locus on chromosome 6q23 influencing fetal hemoglobin levels in adults. *Proc. Natl. Acad. Sci. U.S.A.* 104 11346–11351. 10.1073/pnas.0611393104 17592125 PMC2040901

[B117] Tolhuis B. Palstra R. J. Splinter E. Grosveld F. de Laat W. (2002). Looping and interaction between hypersensitive sites in the active beta-globin locus. *Mol. Cell* 10 1453–1465. 10.1016/s1097-2765(02)00781-5 12504019

[B118] Tsang J. C. Yu Y. Burke S. Buettner F. Wang C. Kolodziejczyk A. A. (2015). Single-cell transcriptomic reconstruction reveals cell cycle and multi-lineage differentiation defects in Bcl11a-deficient hematopoietic stem cells. *Genome Biol.* 16:178. 10.1186/s13059-015-0739-5 PMC4576406 26387834

[B119] Uda M. Galanello R. Sanna S. Lettre G. Sankaran V. G. Chen W. (2008). Genome-wide association study shows BCL11A associated with persistent fetal hemoglobin and amelioration of the phenotype of beta-thalassemia. *Proc. Natl. Acad. Sci. U.S.A.* 105 1620–1625. 10.1073/pnas.0711566105 18245381 PMC2234194

[B120] Wang X. Angelis N. Thein S. L. (2018). MYB – a regulatory factor in hematopoiesis. *Gene* 665 6–17. 10.1016/j.gene.2018.04.065 29704633 PMC10764194

[B121] Weatherall D. J. (2001). Phenotype-genotype relationships in monogenic disease: lessons from the thalassaemias. *Nat. Rev. Genet.* 2 245–255. 10.1038/35066048 11283697

[B122] Weatherall D. J. Edwards J. A. Donohoe W. T. (1968). Haemoglobin and red cell enzyme changes in juvenile myeloid leukaemia. *Br. Med. J.* 1 679–681. 10.1136/bmj.1.5593.679 4966603 PMC1985380

[B123] Wegner M. (1999). From head to toes: the multiple facets of Sox proteins. *Nucleic Acids Res.* 27 1409–1420. 10.1093/nar/27.6.1409 10037800 PMC148332

[B124] Weinberg R. S. Leibowitz D. Weinblatt M. E. Kochen J. Alter B. P. (1990). Juvenile chronic myelogenous leukaemia: the only example of truly fetal (not fetal-like) erythropoiesis. *Br. J. Haematol.* 76 307–310. 10.1111/j.1365-2141.1990.tb07891.x 1709807

[B125] Wienert B. Funnell A. P. Norton L. J. Pearson R. C. Wilkinson-White L. E. Lester K. (2015). Editing the genome to introduce a beneficial naturally occurring mutation associated with increased fetal globin. *Nat. Commun.* 6:7085. 10.1038/ncomms8085 25971621

[B126] Wienert B. Martyn G. E. Funnell A. P. W. Quinlan K. G. R. Crossley M. (2018). Wake-up sleepy gene: reactivating fetal globin for beta-hemoglobinopathies. *Trends Genet.* 34 927–940. 10.1016/j.tig.2018.09.004 30287096

[B127] Wienert B. Martyn G. E. Kurita R. Nakamura Y. Quinlan K. G. R. Crossley M. (2017). KLF1 drives the expression of fetal hemoglobin in British HPFH. *Blood* 130 803–807. 10.1182/blood-2017-02-767400 28659276

[B128] Wolff L. (1996). Myb-induced transformation. *Crit. Rev. Oncog.* 7 245–260. 10.1615/critrevoncog.v7.i3-4.60 9258605

[B129] Xu J. Bauer D. E. Kerenyi M. A. Vo T. D. Hou S. Hsu Y. J. (2013). Corepressor-dependent silencing of fetal hemoglobin expression by BCL11A. *Proc. Natl. Acad. Sci. U.S.A.* 110 6518–6523. 10.1073/pnas.1303976110 23576758 PMC3631619

[B130] Xu J. Sankaran V. G. Ni M. Menne T. F. Puram R. V. Kim W. (2010). Transcriptional silencing of {gamma}-globin by BCL11A involves long-range interactions and cooperation with SOX6. *Genes Dev.* 24 783–798. 10.1101/gad.1897310 20395365 PMC2854393

[B131] Yi Z. Cohen-Barak O. Hagiwara N. Kingsley P. D. Fuchs D. A. Erickson D. T. (2006). Sox6 directly silences epsilon globin expression in definitive erythropoiesis. *PLoS Genet.* 2:e14. 10.1371/journal.pgen.0020014 16462943 PMC1359074

[B132] Zago M. A. Wood W. G. Clegg J. B. Weatherall D. J. O’Sullivan M. Gunson H. (1979). Genetic control of F cells in human adults. *Blood* 53 977–986. 10.1182/blood.v53.5.977.977 373818

[B133] Zeng J. Wu Y. Ren C. Bonanno J. Shen A. H. Shea D. (2020). Therapeutic base editing of human hematopoietic stem cells. *Nat. Med.* 26 535–541. 10.1038/s41591-020-0790-y 32284612 PMC7869435

[B134] Zhou D. Liu K. Sun C. W. Pawlik K. M. Townes T. M. (2010). KLF1 regulates BCL11A expression and gamma- to beta-globin gene switching. *Nat. Genet.* 42 742–744. 10.1038/ng.637 20676097

[B135] Zhu X. Wang Y. Pi W. Liu H. Wickrema A. Tuan D. (2012). NF-Y recruits both transcription activator and repressor to modulate tissue- and developmental stage-specific expression of human gamma-globin gene. *PLoS One* 7:e47175. 10.1371/journal.pone.0047175 23071749 PMC3468502

